# Roman aqueduct maintenance in the water supply system of Divona, France

**DOI:** 10.1038/s41598-023-38655-z

**Published:** 2023-08-04

**Authors:** Gül Sürmelihindi, Cees W. Passchier, Didier Rigal, Andrew Wilson, Christoph Spötl

**Affiliations:** 1 School of Archaeology, University of Oxford, Oxford, UK; 2 Institute for Geosciences, University of Mainz, Mainz, Germany; 3 Inrap GSO, Albasud, impasse de Lisbonne, Montauban, France; 4 School of Archaeology/Faculty of Classics, and All Souls College, Oxford, UK; 5 Institute of Geology, University of Innsbruck, Innsbruck, Austria

**Keywords:** Environmental sciences, Environmental social sciences

## Abstract

Carbonate deposits formed in Roman aqueducts provide a window onto the environment and water management in antiquity. These laminated archives precipitated over a period of decades to centuries and are a potential high-resolution source of unwritten history. However, their use as environmental archives is hampered by local and partial removal during maintenance work in some aqueducts. This apparent problem, however, creates a unique opportunity to study Roman water management. We present the discovery of traces of regular maintenance in carbonate deposits of the Roman aqueduct of *Divona* (Cahors, France). The main objective of this study is to determine the periodicity of local carbonate removal and repairs in this aqueduct. Traces such as tool marks, calcite deformation twins, debris from cleaning and repairs are attested in the deposits as proof of periodic manual carbonate removal by Roman maintenance teams. The δ^18^O profile, recording at least 88 years of deposition, shows that maintenance work was done at intervals of 1–5 years. The undisturbed periodicity of the δ^18^O profile indicates that work was carried out rapidly and never in summer, consonant with the advice of the Roman author *Frontinus* about maintenance of the aqueducts of the city of Rome. Maintenance intervals lengthened and cleaning became less frequent close to the final years of the aqueduct. This change in maintenance policy gives insight into changing local population and socio-economic dynamics in late antiquity.

## Introduction

Water technology in the form of aqueducts was an integral part of Roman culture and one of its most impressive technical achievements. Although the construction and spread of these water supply systems is understood to some extent^1,2^, little is known about their environmental setting, and even less about maintenance during their period of use. Aqueduct channels were commonly encrusted with calcium carbonate deposits that were occasionally removed. These deposits are now a boon to science, as they store information about changes in water flow and chemistry. These changes are in turn linked to natural variations in local rainfall, temperature, vegetation cover and in-channel biological activity with a possible resolution of days to hours over periods of decades to centuries^3–7^. The deposits are also a potential source of information on water management and the chronology of use and modifications^8–11^, providing insight into local population dynamics and socio-economic factors.

Although aqueduct carbonate is a potential high-resolution archive of environmental history directly linked to human settlements, the loss of information due to carbonate removal in antiquity is a deterrent to its use, because parts of the stratigraphy may be lost. Evidence of manual removal of carbonate deposits has been observed in many sites, e.g., the aqueducts of Rome^12,13^, Nîmes^14^, Reims^15^, Béziers^5^, Fréjus^16^, Istanbul^10^ and the Roman water-mill sites of Barbegal^8^ and Saepinum^17^. Local carbonate removal has usually been identified from a single cleaning surface, reflecting an isolated maintenance event. However, some water structures, such as water-mills, needed to be cleaned more frequently than typically capaciously built aqueduct channels^8,17^, because the turbulence created by their mill-wheels led to faster carbonate deposition. Continuous δ^18^O micromilling profiles measured on carbonate from the water gutters that supplied the water-mills at Barbegal (France), confirmed that gutters feeding mills had a different operating schedule from aqueducts used for urban water supply^8^. Truncations discovered in these stable isotope profiles were regular and always coinciding with late summer and early autumn. Hence, it is concluded that the mills were not working continuously, unlike a drinking water supply. We documented regular maintenance traces in the carbonate fragments of the water-mill machinery, interpreted to result from replacement of wooden structures approximately every 5 to 10 years^8^.

In this paper, we present a discovery from the aqueduct of *Divona* (Cahors, France) where exceptionally well preserved multiple periodic cleaning traces in aqueduct carbonate deposits are documented as a first example where the periodicity of cleaning in an urban water supply aqueduct can be established. Our analyses focused on understanding of Roman water management and in particular the frequency of carbonate removal, maintenance strategy and timing of interruptions to the water flow, while providing a strategy for reconstructing the original stratigraphic sequence and providing an example for similar studies of ancient aqueduct carbonates with maintenance traces elsewhere. At the same time, we demonstrate how deposits with traces of major anthropogenic interventions should be examined to improve the reliability and relevance of environmental data from aqueduct carbonate stratigraphy while providing valuable archaeological information^3,4,6–11^. As the potential of aqueduct carbonate studies to contribute to environmental studies becomes increasingly appreciated, our study is needed to identify time gaps during repairs and maintenance that would affect the environmental record provided by the carbonate sequence.

### Divona Cadurcorum (Cahors)

**Figure 1:**
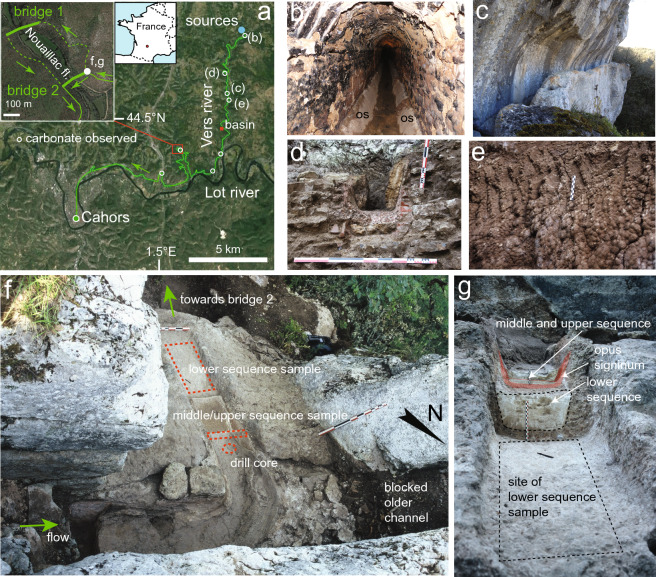
Field aspects of the Divona aqueduct. (**a**) Map of the aqueduct of Divona (Cahors). Carbonate samples were taken just upstream of a bridge on the Nouailhac stream (inset), whose construction shortened the aqueduct course, leaving an abandoned loop along the valley. Locations of other sites where carbonate was observed and mentioned in this article are indicated. (**b**) Masonry channel near the spring. Triangular wedges of ‘*opus signinum*’ on the lower parts of the walls give the channel an intentionally trapezoidal shape. The *opus signinum* is covered by a thin veneer of carbonate. (**c**) Rock-cut section of the aqueduct on a steep cliff. (**d**) Channel with sidewall carbonate where the bottom lacks incrustation. (**e**) Tool marks of cleaning on deposits along the sidewall in a rock-cut channel. (**f**) Location of the main sampling site. In the centre, carbonate deposits with the excavation stages can be seen. (**g**) View of the same deposits from downstream. The satellite image (a) is from Google Maps/Google Earth (data provider: Image Landsat/Copernicus); the drawing program used is Adobe Illustrator 24.2.1.

*Divona Cadurcorum*, a Gallo-Roman city in south-western France, was served by a Roman aqueduct 31.6 km long, bringing water from a source in the Vers valley, 13 km north-east of modern Cahors (Fig. 1a)^18–21^. Discharge is estimated at 11,700 m^3^/day when new and 6800 m^3^/day at the end of use after flow had been constricted by carbonate deposits in the channel^21^. The aqueduct was built between 10 BC and AD 10^19–21^ and was probably active until sometime in the fourth or even the early fifth century AD, since the bath complex in *Divona* that was fed by it fell into disuse around that time^22^. The aqueduct channel was built for long stretches as a rock-cut construction (Fig. 1c) and further as a masonry channel (Fig. 1b). The channel had a trapezoidal cross-section over long stretches (Fig. 1b,d,g). Typically, it is *c.* 32 cm wide at the bottom, widening upward to *c.* 62 cm at 53 cm above the floor (Fig. 1d,g). The trapezoidal shape was intentionally created by a wedge-shaped coating of *opus signinum,* a pinkish/reddish waterproof cement made with crushed terracotta or tile, which thickens towards the bottom within a rectangular channel cross-section.

### Carbonate deposits

The *Divona* aqueduct is encrusted in some places with considerable calcium carbonate deposits, which gave us the opportunity for this study. These deposits form in water structures when the groundwater that supplies them has a high carbonate load that is held in solution by elevated levels of dissolved CO_2_ that exceed atmospheric CO_2_ pressure^3,4,23,24^ (Supplementary Fig. S9). As soon as the groundwater emerges from the subsoil and begins to flow in an aqueduct, with air above the water surface, the excess CO_2_ escapes and excess dissolved carbonate precipitates. Increased turbulence, for example at sharp bends or steep channel sections, results in increased degassing and thus increased carbonate deposition^4^. Most Roman aqueducts were fed by karst springs carrying carbonate-supersaturated water, leading to significant deposits along aqueduct lines^2^. The *Divona* aqueduct was supplied by a karst spring and a branch from the river Vers at the site of a dam that raised the water level^21^.

There is little or no carbonate deposition in the first section of the aqueduct (Fig. 1b), but carbonate deposition increased downstream (Fig. 1d,g). This is a consequence of progressive degassing as the water flows along the channel. Evidence of maintenance was found at several locations in the form of unconformities in the carbonate stratigraphy, some of which show clear tool marks^24^. Figure 1e shows the use of a mattock-like hand tool with a flat blade 3 to 4 cm wide to remove carbonate, while the absence of bottom deposits could be due to preferential cleaning (Fig. 1d). The most prominent site along the aqueduct that shows regular carbonate cleaning, however, is at Nouailhac, 17.2 km from the source (Fig. 1f,g).

## Materials and methods

Analytical work was done as outlined in earlier studies of carbonate deposits in ancient water structures (Supplementary Fig. S8)^3–6,8^. A drill core and a 50-cm long block of carbonate deposits (Supplementary note S1) were taken from the channel of the *Divona* aqueduct at a site named Nouailhac where thick carbonate deposits had been exposed in an excavation in 2003 (44.48185°N, 1.52024°E). The block was cut into slabs using a large-diameter diamond saw with a blade width of 2 mm, losing *c*. 5 mm by cutting and polishing (Supplementary Figs. S1,S2,S3). One slab (B) from the block was cut and two adjacent sub-sections used for further analysis: one was used to make polished thin sections to examine the microstructure and preferred orientation of calcite crystals using transmitted-light microscopy; the other sub-section was polished and used for stable oxygen and carbon isotope analyses which were carried out at the University of Innsbruck (Supplementary Fig. S4). The samples were micromilled at 0.2 mm intervals perpendicular to bedding. The sample powders were analysed using a semi-automated device (Gasbench II) linked to a ThermoFisher Delta V Plus isotope ratio mass spectrometer. Isotope values are reported on the VPDB scale and long-term precision is better than 0.1‰ for both δ^13^C and δ^18^O^25^. When planning the micromilling track, special care was taken to avoid stratigraphic gaps due to unconformities. This was accomplished by carefully comparing the stratigraphy in the drill core and in the collected blocks to select a sample where the stratigraphy was complete.

## Results

### Nouailhac samples

**Figure 2:**
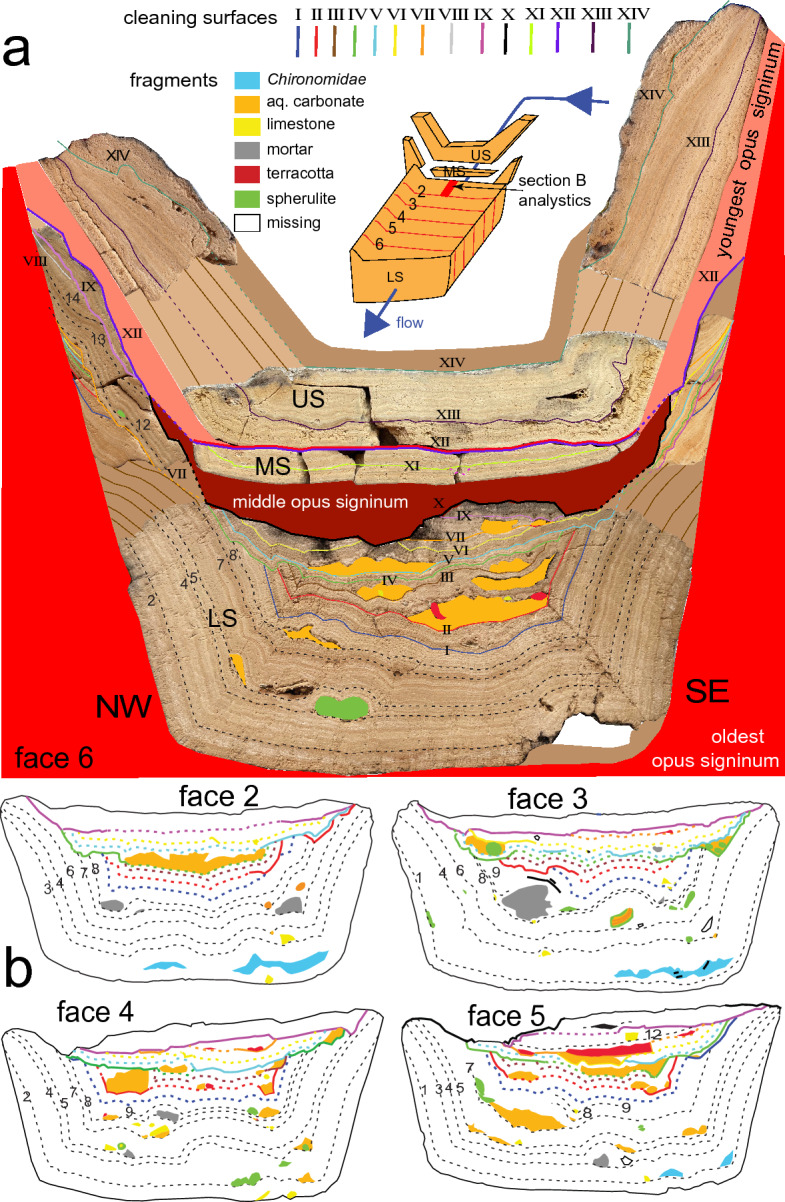
Details of sample stratigraphy, cleaning- and fragment surfaces. (**a**) Reconstruction of the complete sequence at Nouailhac. Lower-, middle-, and upper carbonate sequences (LS, MS, US) are separated by *opus signinum*. MS and US deposits were found as shown, but LS deposits were observed in a block offset about 1 m downstream (Fig. 1f,g). The sample is shown looking upstream to the northeast. Solid brown colours reconstruct missing carbonate layers. The sketch of the block at the top shows relative position of sequences and observation faces. (**b**) Four additional observation faces of the LS block, position as indicated in the sketch in (**a**). Unconformities are interpreted as cleaning surfaces and indicated with coloured lines and Roman numerals. Fragment surfaces are indicated with black dotted lines and Arabic numerals. Coloured fields represent foreign bodies included in the stratigraphy.

**Figure 3:**
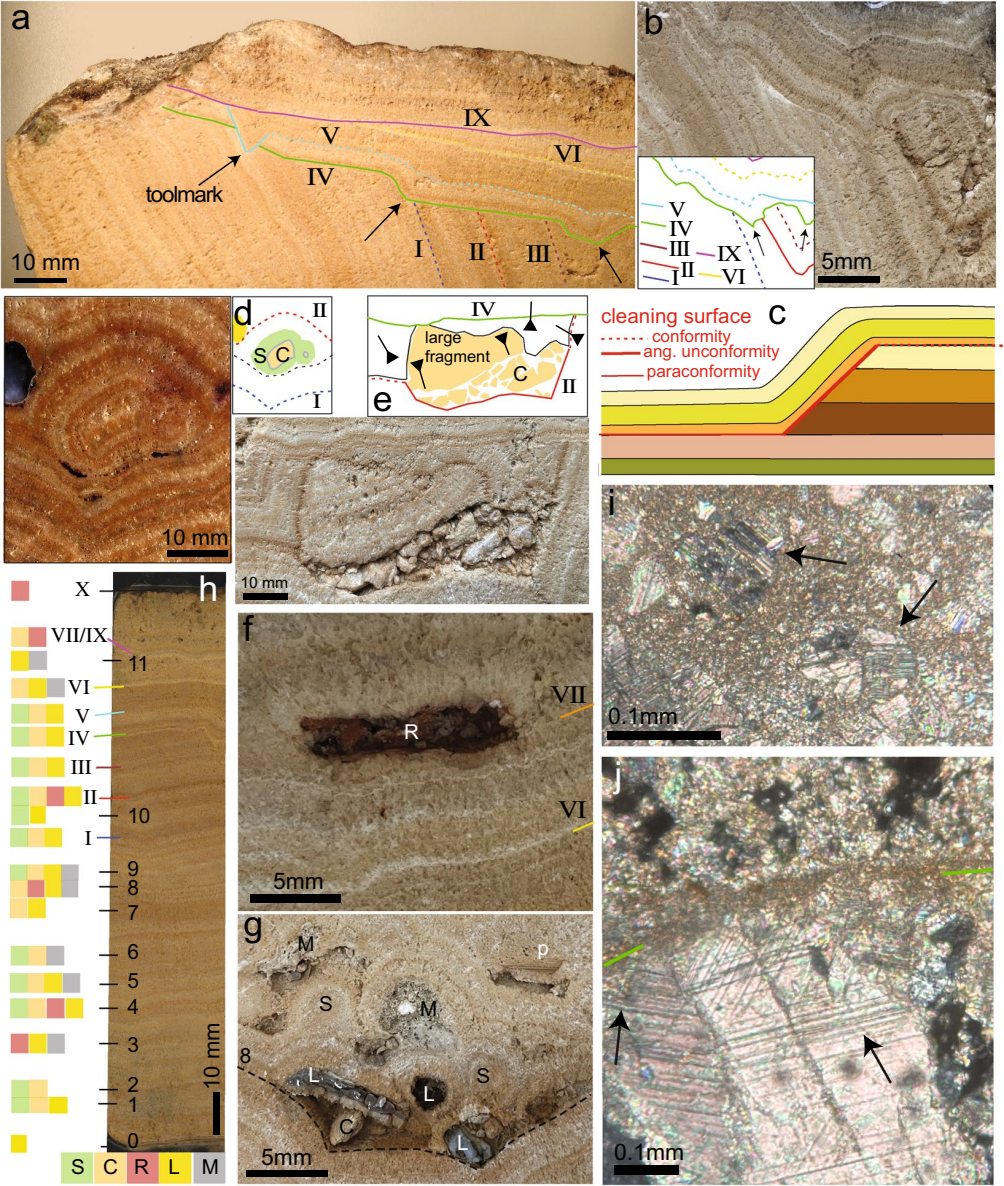
Details of cleaning surfaces (CS) and fragment surfaces (FS). (**a**) Part of observation face 2 with seven CS, and tool marks in CS IV and V (arrows). Unconformities marked as solid lines, and conformities shown as dotted lines. (**b**) Part of face 6 showing seven CS. Arrows indicate tool marks in CS IV. (**c**) schematic diagram of a CS showing a paraconformity, angular unconformity and conformity. (**d–g**) Examples of inclusions and unconformities. (**d**) Spherulite around a fragment of older aqueduct carbonate and rock fragment at left. (**e**) Fragments of older aqueduct carbonate overlain by a dark brown layer. CS IV is visible above, truncating the top of a large fragment. The large fragment was deposited upside down (arrows show younging direction). (**f**) Aggregate of terracotta fragments. (**g**) Diverse inclusions on FS 8 including mortar, spherulites on older carbonate fragments, limestone fragments and a plant imprint (p). (**h**) Distribution of fragments, CS and FS in the stratigraphy of the lower sequence in the drill core. Position of CS and FS are indicated by coloured and black lines and by Roman and Arabic numerals, respectively. Coloured squares at left indicate type of inclusions found on CS and FS. S-spherulite; C-aqueduct carbonate; R-terracotta; L-rock, mostly limestone; M-mortar. (**i,j**) thin section images. Crossed polarised light. (**i**) Crushed calcite crystal fragments with deformation twins (arrows) in micrite covering a CS. (**j**) Deformation twins (arrows) in calcite crystals below CS IV (green line), followed by micrite and broken carbonate fragments.

On the southern, E–W oriented section along the river Lot, the aqueduct made a large detour across the valley of the Nouailhac brook (Fig. 1a inset)^19–21^. An original aqueduct bridge (bridge 1) at a narrowing of the valley was at some later date abandoned and replaced by a bridge to the south (bridge 2, Fig. 1a inset), shortening the channel by some 1.2 km. Since the shortcut reduces the length of the existing aqueduct, the channel across bridge 2 was relatively steep. In the section just upstream of bridge 2, after a sharp rock-cut bend, massive carbonate deposits with a total thickness of 28–30 cm fill the channel almost to the top of the cut (Fig. 1f): probably, the rapid flow and increased turbulence on the steep section after the bend caused solid and dense carbonate to deposit here. The channel has a trapezoidal cross-section formed by a wedge-shaped *opus signinum* lining (Figs. 1g and 2a). Two renewal coatings of *opus signinum* separate the carbonate stratigraphy into a lower-, middle-, and upper sequence (Figs. 1g and 2a). A 50 cm-long block of lower sequence carbonate (Supplementary note S1) had detached from the walls and was collected for this study (Figs. 1f and 2, Supplementary Fig. S1). The middle- and upper sequences were collected in a 7-cm wide strip of the adjacent remaining channel fill, together with sidewall deposits (Fig. 2a, Supplementary Fig. S5). A drill core was taken approximately 40 cm upstream from the block to obtain a fresh, unweathered sample of the entire stratigraphy (Figs. 1f and 3h).

### Carbonate stratigraphy

The block of lower sequence deposits was cut lengthwise into 6 slabs of about 8 cm width. The saw cuts were polished and used for observation as faces 1–6 (Fig. 2, Supplementary Fig. S3). Lamina couplets usually have irregular thickness and highly variable colour change (Fig. 3a, b) in contrast to the highly periodic annual layers and bicolour fabric, typical of aqueduct carbonates in the eastern Mediterranean where seasonal variation in rainfall is more pronounced^3,4^, but similar to other western Mediterranean examples^5–7^. The detection of annual layers in hand specimens is therefore difficult. The microfabric shows an alternation of calcite microsparite and micrite (Fig. 3j, Supplementary Fig. S7a) and differs from the more crystalline and dominantly elongate calcite crystals of samples from the eastern Mediterranean^3,4^.

### Unconformities and inclusions

The Nouailhac carbonate deposit shows many interruptions or ‘unconformities’ throughout the stratigraphy, where layers are cut and overlain by undisturbed younger layers (Fig. 2). In thin section, unconformities are marked by truncated rhomboidal sparite crystals covered by a layer of micrite along a sharp, serrated boundary (Fig. 3j, Supplementary Figs. S7e–h). The sparite crystals at unconformities commonly show deformation twins at the contact (Fig. 3j)^26^, while the micritic layer contains angular calcite crystal fragments with deformation twins (Fig. 3i). At least 14 unconformities were identified in the Nouailhac carbonate stratigraphy. They are narrowly spaced in the top of the lower sequence but are missing at the bottom (Fig. 2). The upper sequence has only two unconformities.

Besides unconformities, numerous inclusions are trapped at different levels of the lower sequence. These include fragments of older aqueduct carbonate and mortar, angular bedrock limestone pieces, and small terracotta fragments (Figs. 2, 3d–g). Fragments lie isolated in pockets on the surface of unconformities, especially in cavities at the bottom of the channel where particles could easily settle (Figs. 2, 3d–g). Some isolated particles developed into spherulites as they were rolled along in the channel (Fig. 3d,g, Supplementary Fig. S4). Fragments and spherulites can form an unconformity ‘zone’ separating individual layers (Figs. 2 and 3e).

### Stable isotope and stratigraphic profile

**Figure 4:**
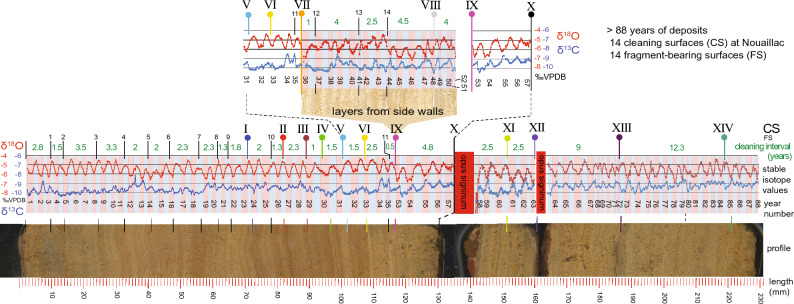
Full stable isotope profile of the Nouailhac sample, linked to the sample stratigraphy. Positions of cleaning surfaces are marked with vertical coloured pins and labelled with Roman numerals. Fragment surfaces are marked with vertical black lines and Arabic numerals. The estimated intervals between cleaning surface and fragment surface in years are shown by the green numbers. Blue and red alternating bars behind the isotope profile indicate inferred winter and summer seasons, respectively, with sequential year numbers. A corresponding section of the drill core stratigraphy is shown at the bottom. An additional section of stratigraphy, not present in the drill core, was found in sidewall deposits (Fig. 2a) and is shown above the main profile as an inset. This stratigraphy was removed in the drill core section during cleaning event IX as indicated. The relationship of this additional stratigraphy with the main profile is shown by repetition of short sections of the main stratigraphy in the inset for years 31–57.

Figure 4 shows a continuous stable C and O isotope record with unconformities indicated by Roman numerals, established by cross-correlation of the block samples with the drill core (Figs. 2 and 3). The δ^18^O curve shows a pronounced cyclicity comparable to that of eastern Mediterranean aqueduct carbonate (Fig. 4)^3,4,6,9^. This cyclicity is mainly attributed to temperature-dependent kinetic isotope fractionation during carbonate precipitation reflecting seasonally changing air and water temperatures^3,4^, whereby less negative δ^18^O values represent cooler periods and vice versa. The δ^18^O curve therefore allows recognition of summer and winter seasons with a resolution up to one month, as marked in Fig. 4. Unlike eastern Mediterranean aqueduct carbonate^3–6,9^, there is only a weak anti-correlation between δ^18^O and δ^13^C, some years showing a covariation, and no correspondence with the layering visible in hand specimen. In the drill core and the slabs used for analyses (Supplementary Fig. S4), only 40 annual δ^18^O cycles were counted in the lower sequence (Fig. 4), but an additional stratigraphy of 17 layers was found on slab faces 3–6 (Fig. 2, Supplementary Fig. S3) and in the thin side deposits (Fig. 2a). With 31 annual layers recognised in the middle- and upper sequence, evidence therefore exists for at least 88 years of carbonate deposition in the aqueduct (Fig. 4).

### Changes at the top of the sub-stratigraphies

The final sequences of the three sub-stratigraphies of the Nouailhac sample show different features. The top of the lower sequence, above unconformity IX, shows a gradual increase in δ^18^O and δ^13^C values in the last 4 cycles (Fig. 4). Carbonate becomes more porous between IX and X and contains abundant impressions of algal filaments (Supplementary Fig. S7b).

No change in fabric was observed at the top of the middle sequence, but a change in the stable isotopic composition follows unconformity XI, with more negative δ^18^O and more positive δ^13^C values in the 2.5 years before surface XII and the second re-plastering event. Similar changes in stable isotopes were observed after unconformity VII and fragment surface 14 in the lower sequence (Fig. 4).

The top of the upper sequence shows a fabric transition similar to the top of the lower sequence towards a more porous carbonate fabric, albeit with increased clay content, but without algal filaments and without a significant change in the stable isotope pattern (Fig. 4, Supplementary Fig. S7c).

## Discussion

### Anthropogenic impact and cleaning surfaces

The Nouailhac carbonate deposit is exceptional for its large number of unconformities. These are evident where younger layers overlie truncated older layers, which in geology is called an angular unconformity (Fig. 3b, c). Angular unconformities grade laterally into surfaces parallel to layering, which form either because the carbonate has split along the layering during cleaning, with material missing (paraconformity), or represents the final carbonate-water contact (conformity). Only in the case of conformity surfaces is the stratigraphy continuous. Unconformities mark sites where previously deposited material has been removed.

Deformation twins in calcite crystals formed at unconformities and in the overlying micrite are signs of physical damage: calcite crystals develop crystallographic twins when they are put under pressure or respond to shock (Fig. 3i, j, Supplementary Fig. S7g–j)^26–28^. Locally irregular cm-size steps in unconformities have the form of tool marks (Fig. 3a, Supplementary Fig. S3). Taken together, these observations indicate that the unconformities did not develop naturally but are due to the manual removal of carbonate. We therefore use the term ‘cleaning surface’ to refer to the stratigraphic levels immediately overlying the unconformities. In most cases, these levels are marked by the presence of persistent thin white micritic bands with broken crystal fragments, most likely caused by refilling of the aqueduct with water after it had been closed for a short time and had dried out, redistributing the remaining carbonate debris and dust from cleaning, followed by nucleation of micrite (Fig. 3b, i, j).

### Debris fragments

Foreign inclusions of older aqueduct carbonate, spherulites, terracotta, grey mortar, and limestone bedrock (Figs. 2 and 3d–g) lie on the cleaning surfaces but also in the first layers of the lower sequence where cleaning surfaces are absent. Aggregates of angular old carbonate fragments are most common. Larger fragments can be recognised by their internal layering pattern as parts of the underlying stratigraphy (Fig. 3e). The crushed crystals at the edges of these fragments (Fig. 3i) suggest that they are traces of human activity upstream of Nouailhac, where debris of the chipped-off carbonate was left in the channel and was subsequently washed downstream. Terracotta fragments with a diameter of 1–2 mm are concentrated in small pockets, mainly on cleaning surfaces II and VII (Figs. 2 and 3f, Supplementary Figs. S2, S3 and S6). Most likely, these represent storage of crushed terracotta used for preparation of *opus signinum* for repair inside the channel upstream of Nouailhac, later washed downstream. Fragments of grey mortar and limestone (Fig. 3g) may represent mortar spilled during repair work, and broken rock fragments spilled into the channel during preparation of mortar. Most fragments are bare, probably because they were transported rapidly over a short distance and covered by younger deposits (Figs. 2 and 3h). Fragments that travelled a longer distance were gradually covered by new calcite and developed into spherulites (Fig. 3d, g).

Fragments present in the first 8 cm of the lower sequence are not randomly distributed but occur periodically in at least 14 horizons, which we have termed fragment surfaces (Fig. 2, Supplementary Figs. S2, S3 and S6). The main fragment surfaces and their relative chronology are marked in Figs. 3h and 4. The types of fragments on fragment surfaces are similar to those on cleaning surfaces, and at least some contain terracotta fragments (Figs. 2b, 3h). We therefore think that all stratigraphic levels containing fragments correspond to cleaning or re-plastering repair work in the aqueduct channel upstream of Nouailhac. Apparently, the maintenance team started cleaning and repairing some upstream sections of the aqueduct shortly after the channel across the bridge at Nouailhac was first put into service but waited 23 years before the channel was cleaned of carbonate for the first time at the Nouailhac site. All stratigraphic levels containing fragments are therefore thought to correspond to cleaning or re-plastering repair work in the aqueduct channel.

### Periodic cleaning

Based on the seasonally resolved δ^18^O profile (Fig. 4), the time interval between cleaning surfaces in the entire sequence can be determined. The lower- and middle sequence are found to have an interval of 1–5 years, with a mean of 2.8 years (Fig. 4). Apparently, a regular cleaning regime was introduced. The time interval separating fragment surfaces in the first 8 cm of the lower sequence is similar but slightly shorter than cleaning surfaces in the upper part of the lower sequence and in the middle sequence at Nouailhac, probably because more cleaning and repair events are recorded over a longer section of channel (Fig. 4). The upper sequence is different, with only two cleaning episodes at intervals of 9.5 and 12.5 years. This could be due to a different cleaning strategy or, more likely, to population decline and reduced water demand, or because fewer resources were available for maintenance. It is obvious that maintenance in the later Roman period decreased in the last years before the aqueduct was abandoned.

### Frequency and time of cleaning

Interruptions in water supply of more than one month are visible as truncations in the seasonal δ^18^O profile, as seen in samples from the watermills of Barbegal^8^. In the Nouailhac profile, there are no such truncations that correspond to cleaning surfaces in the stratigraphy (Fig. 4). Therefore, the cleaning work, which involved the interruption of the drinking water supply of the city of *Divona*, was carried out in a very short time, less than a month. However, truncations implying longer interruptions were observed at the two re-plastering events, as discussed below.

Cleaning surfaces in Fig. 4 can be grouped according to where they intersect the δ^18^O curve, interpreted in terms of seasonal temperatures. Combining 14 cleaning- and 14 fragment surfaces, 13 events fall in autumn, 10 in spring, 5 in winter, and none in summer. Interestingly, this pattern fits the recommendations of *Sextus Julius Frontinus* (AD 40–103), *curator aquarum* of the city of Rome, to avoid cleaning and repair work in the summer, when demand for water is greatest (Supplementary note S2, Supplementary Fig. S2).

### Depth of cleaning

In the lower sequence, the first 8 cm of carbonate, representing 23 years of annual deposition, are devoid of cleaning surfaces, while 9 cleaning surfaces at the top of the lower sequence and in its preserved side deposits lie close together and are partly overlapping, cutting out parts of earlier cleaning surfaces and subsequent growth (Fig. 2a, Supplementary Fig. S3). Cleaning was apparently done regularly and down to the approximate level of previous cleaning or just above, without cutting deeper. Although the lack of cleaning in the first 8 cm of the lower sequence could be attributed to the negligence of the maintenance crew, it is more likely that the crew was not aware of the true depth of the original new channel and of the thickness of remaining carbonate above the *opus signinum* during the first cleaning at Nouailhac. In the event of damage to the waterproof *opus signinum* lining of the channel, re-plastering would have been necessary, which would have interrupted the water supply for an extended period, and the maintenance team may have tried to avoid this. During subsequent cleaning, care was therefore taken not to cut deeper than during the initial cleaning. Alternatively, the depth of the original channel was known, but full cleaning was not deemed necessary to obtain sufficient discharge; also in this case, cleaning to the approximate earlier levels was maintained. Regardless of the reasons, the partial cleaning pattern at Nouailhac is fortuitous because it preserves evidence of maintenance that would have been lost if all carbonate had been removed.

### Significant events in the sequence

Some events recorded in the stratigraphic sequence warrant further discussion. After 35 years of deposition and six cleaning episodes in 12 years, cleaning episode VII changed the shape of the channel by removing not only bottom deposits, but also part of the NW wall deposits (Fig. 2a). The stable isotope signal shows a significant change after VII, with δ^18^O decreasing and the δ^13^C signal flattening (Fig. 4). This change is accompanied by a change in the type of lamination, with more pronounced bimodal laminae couplets after VII (Fig. 4). A similar rapid decrease in δ^18^O, with a rapid increase in δ^13^C, occurred after fragment surface 14 (Fig. 4), where δ^18^O and δ^13^C only returned to their original values after some years. Evidently, these changes are due to altered flow conditions in the channel because of human intervention, possibly due to an increased cross-sectional area of the partially cleaned channel providing more space for faster flow and higher discharge.

Apart from regular cleaning, two events of re-lining with *opus signinum* in the Nouailhac deposits, separating the lower-, middle- and upper sequences, represent two major periods of interrupted water flow during the activity of the *Divona* aqueduct, marked by truncations in the seasonal stable isotopic profile (Fig. 4). How long these interruptions lasted cannot be estimated without dating: each interruption could have lasted at least a few months or even up to several years.

There is evidence that the water supply faced problems some years before the first re-plastering. After cleaning surface IX, year 53 shows a similar microfabric and isotope signal to earlier deposits but changes in year 54 until the first re-plastering at X after year 57 (Fig. 4). Increased porosity (Fig. 3h) and the abundant presence of algal filaments at the top of the lower sequence after year 54 (Supplementary Fig. S7b) indicate changed conditions of carbonate deposition and enhanced biological activity in the channel. A structural problem such as missing cover stones or damage to the aqueduct vault could have allowed daylight to reach the channel giving rise to algal growth and a gradual transition from solid microsparite to porous micrite, resembling tufa. The increase of δ^18^O and δ^13^C can be explained by increased evaporation, degassing, and algal growth, and possibly a reduced discharge (Fig. 4). Year 57 is marked by strong instability of the isotope curves. These observations suggest low or no maintenance and reduced water quality from year 55 until water flow was interrupted for cleaning and re-plastering in year 57 (Fig. 4). Repair of the Nouailhac channel may have occurred in year 58, with only a short delay, or may have taken longer if repairs were extensive. In any case, re-plastering seems to have happened in response to damage to the channel, and the aqueduct was put back into use during the winter/cooler season.

After the first re-plastering/renewal, carbonate formed over 5 years until a sudden interruption of the water supply, possibly due to some disorder, followed by re-plastering. This time, re-plastering was only along the sides of the channel and not at the bottom, suggesting that it was only meant to optimise the shape of the channel after some deposits from the sidewall were removed, as seen in unconformity XII in Fig. 2a. The bottom of the channel was not touched at this stage but was contaminated with a thin smear of *opus signinum* that caused the separation of the middle- and upper sequences. The second re-plastering coincides with a time gap of at least several months, as recorded by the truncated δ^18^O curve.

The topmost part of the carbonate sequence after cleaning episode XIV and layer 85 shows a fabric change to porous micrite as at the top of the lower sequence (Figs. 3h and 4). No abnormality in the stable isotope pattern is observed, but the top of the sample contains abundant clay mantling micrite, suggesting slope runoff entering a damaged channel. This indicates that the aqueduct carried poor quality water under sub-optimal conditions for at least three years before ceasing operation altogether. It was either not possible to re-activate the aqueduct as had been done on two earlier occasions, or no longer necessary.

The stratigraphy at Nouailhac provides evidence of human interactions with the water supply of *Divona*. The sudden start of regular cleaning after year 23, the two re-plastering events, the change in frequency between cleaning events from the middle- to the upper sequence, and the signs of poor maintenance at the top of the lower- and upper sequences provide evidence for changes in cleaning strategy, and in the case of deteriorating maintenance and longer intervals between cleaning episodes, possible socio-economic stress. Longer intervals between cleaning episodes might reflect a different maintenance regime, a new overseer of the public slaves used on the aqueduct, instability in *Divona*, or declining population of the city. Cleaning episode XIV is also revealing: its position 3–4 years before the final abandonment of the aqueduct testifies to the fact that there was still an effort to continue using the aqueduct and that the final demise of the aqueduct was relatively swift, possibly due to a sudden economic or political event. The insights gained from studying the carbonate deposits in the *Divona* aqueduct can thus be combined with archaeological data from the surrounding area to shed light on the socio-economic conditions in *Divona* in late antiquity.

### Age of the deposits

Archaeological evidence suggests that the *Divona* aqueduct operated in a period from around the beginning of the first century AD until sometime in the fourth or the early fifth century AD^19–22^. There is no direct evidence for the date of its abandonment, which is inferred from the disuse of the city’s public baths, thought to be in the fourth or early fifth century, but hard dating evidence is lacking. The bridge at Nouailhac, where the carbonate sample was taken, belongs to a phase at some unknown date after the aqueduct’s initial construction, in which the course of the aqueduct was shortened to cut off a loop up one side of the valley and down the other. Since the carbonate deposits at Nouailhac represent only 88 years of operation (Fig. 4), with possibly a few extra years missing during re-plastering events, the logical implication is that the Nouailhac bridge was built less than 100 years before the aqueduct’s final abandonment, putting its construction sometime in the third or first half of the fourth century AD. The carbonate deposits would thus date from the third or fourth century, with the final years possibly as late as the early fifth century AD. This suggests a considerable change in the fortunes of *Divona* over the course of the late Roman period: in the first half of the period covered by our carbonate sample, major works of construction were carried out, involving a new aqueduct bridge shortening the channel trajectory, and channel maintenance was well organised during the first 54 years after the construction of the Nouailhac bridge. After that—so in the late third century or the fourth century—we see signs of deterioration until the aqueduct went out of use and could not be rehabilitated. If the Nouailhac bridge was built in the early third century, the changes in the deposits implying deteriorating water quality at the end of the lower sequence might reflect one or more of the various socio-economic troubles recorded after the middle of the third century—including the Plague of Cyprian, the secession of the Gallic Empire, and Alemannic invasions, some of which reached as far as this area of Gaul^29–33^. That scenario would imply a relatively early abandonment of the aqueduct, in the fourth century AD. Alternatively, since construction of the Nouailhac bridge is unlikely during the troubles of the 250s–270s, construction in the early fourth century, perhaps under Constantine, is also a plausible scenario; and in that case the carbonate sequence would reflect maintenance throughout much of the fourth century, with a deterioration in both maintenance and water quality until eventual abandonment in the later fourth century or even the early fifth century.

The layers of the Nouailhac carbonate stratigraphy are like pages in a book of ancient water management: they document the constant efforts for water supply, cleaning, maintenance, and re-plastering of an aqueduct. A detailed analysis of anthropogenic interventions in the water supply, as was done for the *Divona* aqueduct, can be used in archaeo-hydrological studies of carbonate deposits to obtain not only valuable archaeological information but also reliable environmental data from antiquity.

## Conclusion

The aqueduct of *Divona* shows evidence for regular cleaning, mostly without significant interruption of the water supply and, as recommended by *Frontinus*, never in the summer season. The observations made provide new tools to recognise cleaning surfaces in carbonate deposits of ancient water systems. Unconformities linked to deformation twins and tool marks are evidence for carbonate cleaning. Similarly, fragments of carbonate and terracotta concentrated on certain horizons are indications for upstream cleaning. Increasing porosity, clay content and biological activity in carbonate, linked to a decrease in cleaning frequency, can indicate a decrease in overall maintenance. Regular maintenance can be taken as evidence for a well-structured organisation of an urban site, while indirectly, socio-economic stress may result in less regular maintenance or a total lack thereof.

## Supplementary Information

Supplementary Information.

## Data Availability

Data needed to evaluate the conclusions in the paper are present in the paper and/or the Supplementary Materials. The stable isotope results analysed during the current study are available from the corresponding author on reasonable request.
