# Supplementary material for: Roman aqueduct maintenance in the water supply system of Divona, France
Source: Sci Rep. 2023 Aug 4;13:12035. doi: 10.1038/s41598-023-38655-z (PMC10403618; doi:10.1038/s41598-023-38655-z)
Supplement: Supplementary file 1 — Supplementary Information. [file 41598_2023_38655_MOESM1_ESM.docx]

Supplementary Information

The art of Roman aqueduct maintenance: the water supply system of *Divona*, France

Gül Sürmelihindi^1^, Cees W. Passchier^2*^, Didier Rigal^3^, Andrew Wilson^4^, Christoph Spötl^5^

^1^ School of Archaeology, University of Oxford, Oxford, UK

^2^ Institute for Geosciences, University of Mainz, Germany

^3^ Inrap GSO, Albasud, impasse de Lisbonne 82000 Montauban

^4^ School of Archaeology / Faculty of Classics, and All Souls College, Oxford, UK

^5^ Institute of Geology, University of Innsbruck, Austria

**Note S1 - the samples**

This appendix gives additional details of observations made on the main carbonate sample block of Nouailhac, Cahors, France (Fig. S1). This large block, collected at the upstream part of an aqueduct bridge across the Nouailhac brook at 44° 28.911' N, 1° 31.215' E, represents the full lower sequence of the local aqueduct carbonate stratigraphy. Since the block was ca 50 cm long, it was possible to cut it into seven slabs, and to study the stratigraphic succession, included fragments and unconformities over the entire length of the block. Material loss in the saw cuts was 3-4 mm. Slabs are labelled A-G and sawcut surfaces of the slabs are labelled 1-6 (Fig. S1). For example, the surface of sawcut 5 visible on slab F would be labelled 5-F and shows very similar features to surface 5-E since both are separated by only 3 mm. Figures S2 and S3 below show the actual observations, which are presented in abbreviated form in Fig. 2 in the paper.

Besides fragments, several slabs including B, C and D contain layers of *Chironomidae* tubes produced by colonies of these freshwater fly larvae. Interestingly, these colonies occur only on the south-eastern side, on the inside of the bend of the channel at Nouailhac; probably the flow velocity was lower on the inner side behind the bend of the channel where it created favourable conditions for them to grow (Fig. S3).

Foreign inclusions and spherulites were deposited in cavities made during cleaning but are also deposited in cusps formed by irregular growth that created an uneven, bulbous growth surface. Such bulbous growth is common in aqueduct carbonate and speleothems in general and has also been observed to capture spherulites and debris in some other aqueducts.

Figure S4 shows cross-sections of slab B, which was cut up for analyses. Fragments and spherulites visible on these faces were used in Figure S5. The position of tracks of stable isotope micromilling are shown.

Fig. S5 shows the sidewall deposits of the channel above the lower sequence block; these deposits are an integral part of the lower sequence (Fig. 2a) but were lost at the site where the large block sample was taken; we managed to collect them about 1 m upstream, at the site of the drill core (Fig. 1f). The sidewall deposits are important because there was less cleaning here; parts of the lower sequence stratigraphy are preserved here that were lost by cleaning in the main block, especially years (36)-(51). The carbonate deposits on the sidewalls are much thinner than those at the bottom of the channel (Fig. 2a), partly because of a lower deposition rate and partly because of the fluctuating water level. Therefore, it is difficult to reconstruct the stratigraphy of these sidewall deposits: micromilling for stable isotope analysis requires a minimum thickness of about 0.1mm per sample, which is on the order of the annual layer thickness at the top of the sidewall deposits. On the other hand, the lower parts of the sidewall deposits show continuous stratification, including all cleaning surfaces, so that we are confident that no stratigraphy was lost here.

Figure S6 shows a summary of the observations on fragments and spherulites observed in the lower sequence (Figs. S3). This summary, again, was used to construct the abbreviated presentation of fragment types in Figure 3h. Notice that besides the 14 fragment surfaces mentioned in the paper, there are stratigraphic horizons where single spherulites are found in some sections (labelled 3a, 4, 7a etc.) Spherulites can settle on any stratigraphic level, and single isolated spherulites are therefore not considered to represent separate fragment surfaces.

Figure S7 shows a selection of microscopic observations in thin sections made of slab B.

**Paraconformities and unconformities (Fig. 3c).** Paraconformities are difficult to distinguish from conformities but can be identified in larger sections where they can be seen to grade into angular unconformities (Fig. 3c). Paraconformities would also create truncations in the typical sigmoidal seasonal pattern of the δ^18^O profile. Such truncations occur when water flow was interrupted and resumed in different times of the year, either after a few months or after several years in a different season (Fig.4. cleaning surfaces X, XII). If the flow was continuous or interrupted for less than a few weeks, there is a conformable sequence (Fig. 4).

Unconformities in the lower sequence show a tendency to asymmetry: excavation is commonly deeper on the south-eastern side of the channel (the righthand side in Fig. 2; Appendix, Fig. S3). This asymmetry can be explained by the fact that maintenance workers had to operate in the confined space of the aqueduct and carbonate removal was easier on the opposite side of their preferred working hand. If the workers were right-handed, they probably worked looking upstream.

**Note S2 - Frontinus quotation**

Quotation from the work of Sextus Julius Frontinus, "De Aquis urbis Romae", in the original latin, with a translation.

Fron., Aq., 120–122: ‘(120) Nascuntur opera ex his causis: aut impotentia possessorum quid corrumpitur aut vetustate aut vi tempestatum aut culpa male facti operis, quod saepius accidit in recentibus. (121) [...] Minus iniuriae subiacent subterranea nec gelicidiis nec caloribus exposita. Vitia autem eiusmodi sunt, ut aut non interpellato cursu subveniatur eis, aut emendari nisi adverso non possint, sicut ea quae in ipso alveo fieri necesse est. (122) Haec duplici ex causa nascuntur: aut enim limo concrescente, qui interdum in crustam indurescit, iter aquae coartatur, aut tectoria corrumpuntur, unde fiunt manationes quibus necesse est latera rivorum et substructiones vitiari. [...] Refici quae circa alveos rivorum sunt aestate non debent, ne intermittatur usus tempore quo praecipue desideratur, sed vere vel autumno et maxima cum festinatione, ut scilicet ante praeparatis omnibus quam paucissimis diebus rivi cessent.’

‘The necessity of repairs arises from the following reasons: damage is done either by the lawlessness of abutting proprietors, by age, violent storms, or by defects in the original construction, which has happened quite frequently in the case of recent works. (121) [...] The underground portions, not being subjected to either heat or frost, are less liable to injury. Defects are either of the sort that can be remedied without stopping the flow of the water, or such as cannot be made without diverting the flow, as, for example, those which have to be made in the channel itself. (122) These latter become necessary from two causes: either the accumulation of deposit, which sometimes hardens into a crust, contracts the channel of the water; or else the concrete lining is damaged, causing leaks, whereby the sides of the conduits and the substructures are necessarily injured. [...] Repairs to the channel itself should not be made in the summer time, in order not to stop the flow of water at a time when the demand for it is the greatest, but should be made in the spring or autumn, and with the greatest speed possible, and of course with all preparations made in advance, in order that the conduits may be out of commission as few days as possible.’ (Translation: Charles E. Bennett, Loeb edition, 1925)

**Figure S1. Nouailhac sample block**


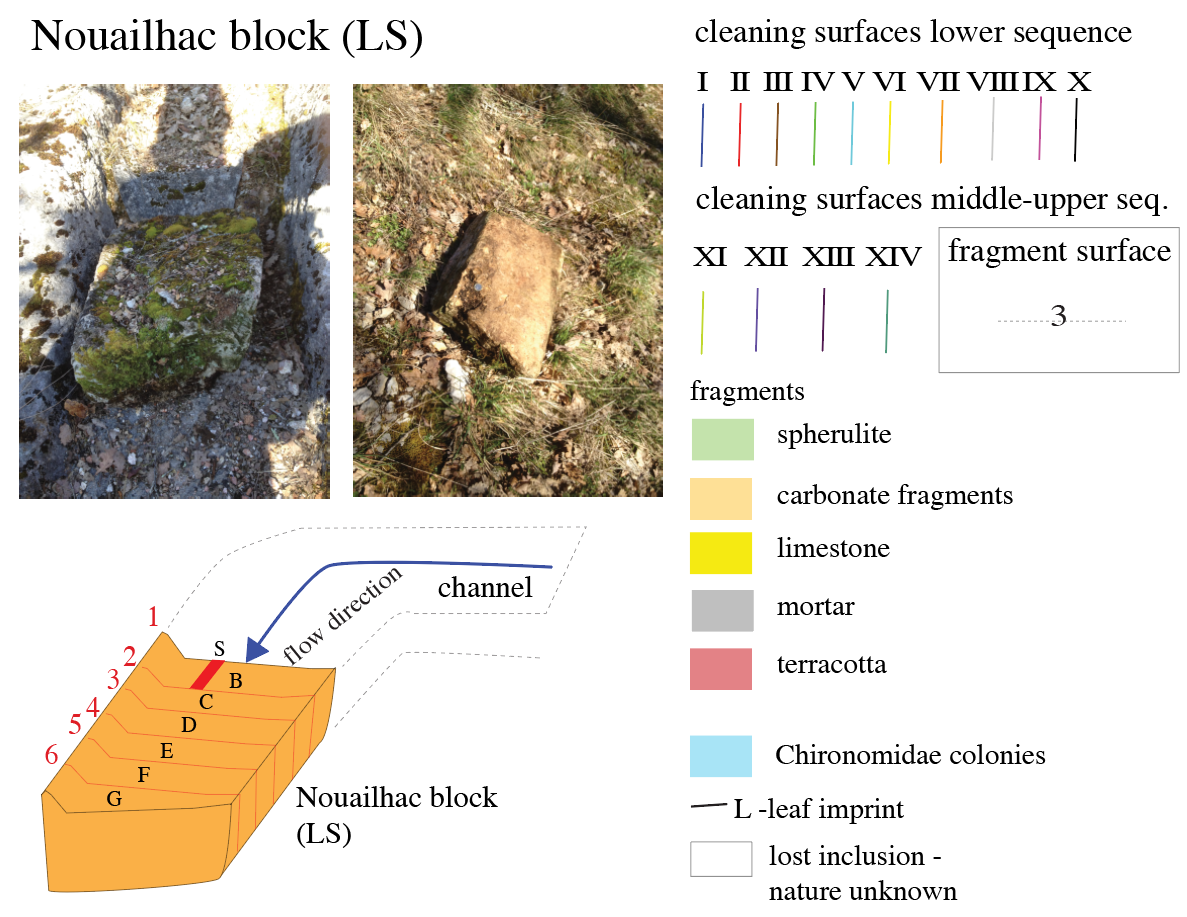


**Fig. S1**. The large block of the lower sequence collected at Nouailhac in its original position (top left) and seen from below (right). The method of sectioning the block is shown below. Letters refer to carbonate slabs, numbers to the surfaces between slabs. Faces of individual slabs facing each other are therefore labelled 2-C and 3-C, 3-D and 4-D etc. In the paper we only give the numbers of the observation faces. In this Appendix and in the paper, all surfaces of carbonate slices are shown looking upstream, from G to A. Some photos and diagrams have been reversed for this purpose, with an indication where this has been done. The legend for colors used in Figures is shown at right.

**Figure S2. Lower sequence slab faces with interpretation**


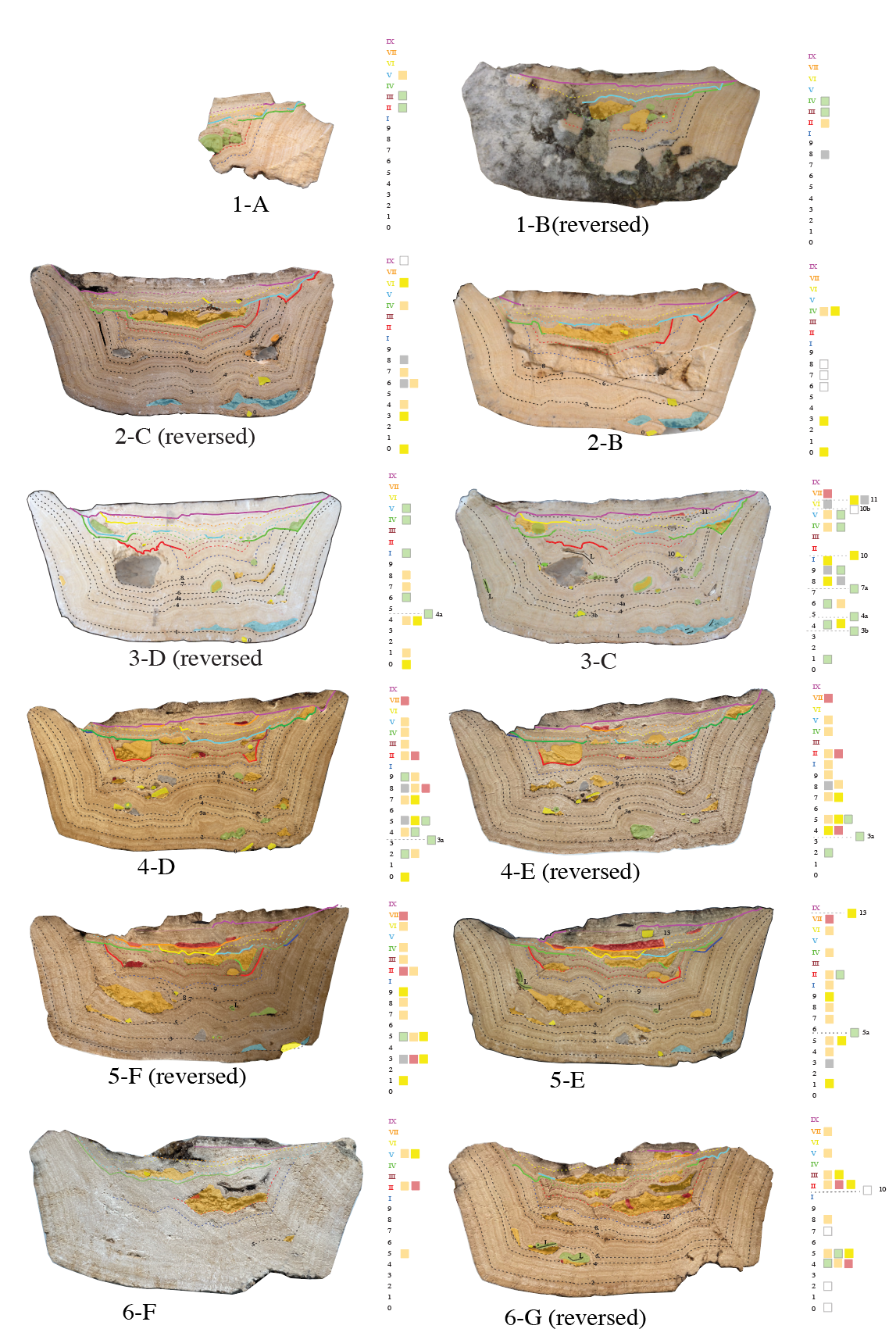


**Fig. S2**. Lower sequence slab faces with interpretation of cleaning surfaces as colored lines, and fragments or fragment-accumulates as transparent colored fields. This is a low-resolution overview - details in Fig. S3. Some photos are shown in mirror image (reversed) for easy comparison, as indicated. View looking upstream. Slab B was cut up and used for all geochemical analyses and thin sections. Color markings beside the slabs summarize fragments found on individual surfaces. Black dashed lines indicate stratigraphic surfaces where fragments were found (fragment surfaces). High resolution images of the slab faces are given in Fig S3.

**Figure S3. Lower sequence slab faces with interpretation - details**

*
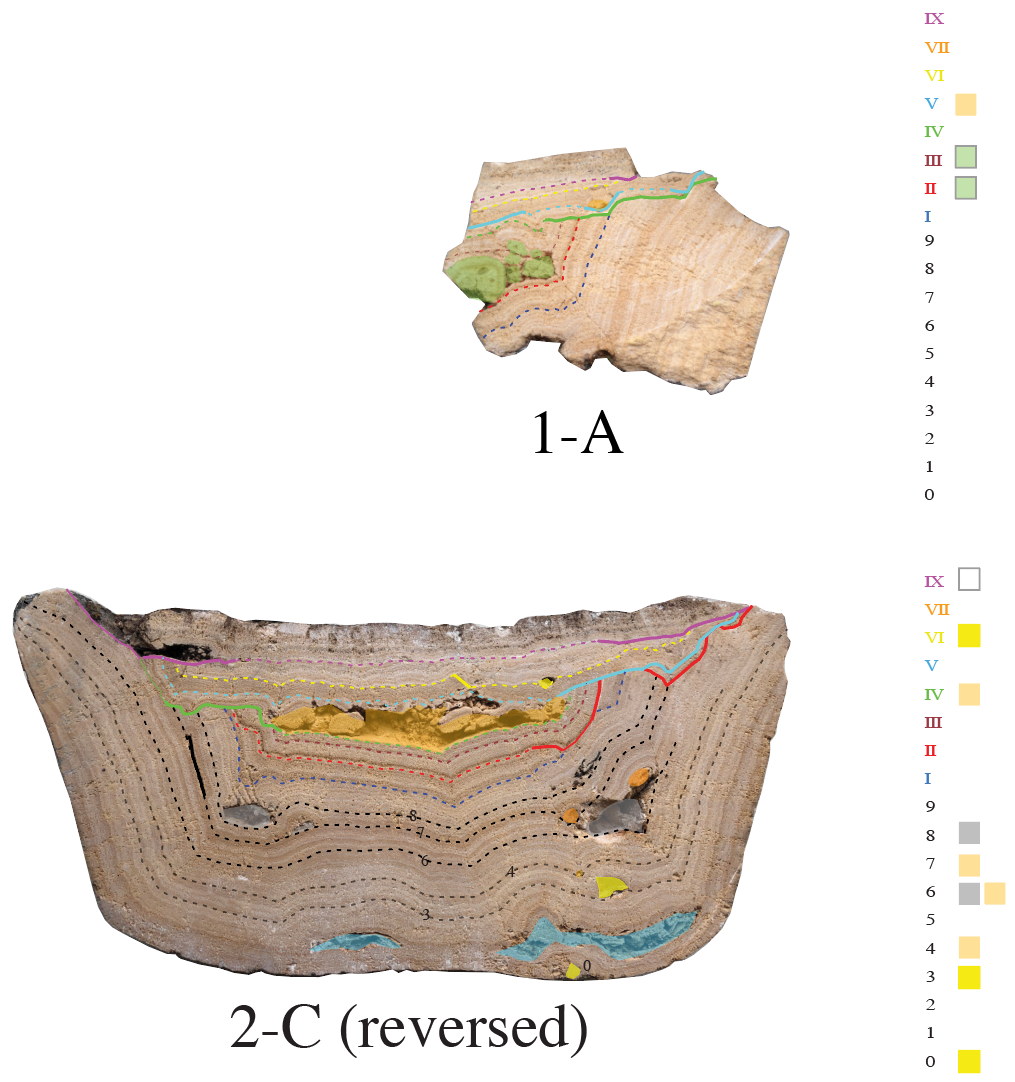
*

**Fig. S3.** High-resolution images of slab faces shown in Fig. S2 with interpretation of cleaning surfaces as colored lines, and fragments or fragments accumulates as transparent colored fields. Some photos are shown in mirror image(reversed) for easy comparison. View looking upstream. Surfaces are distinguished in three categories: solid lines - angular unconformity; thin lines - paraconformity; dashed lines - conformity. Legend for colors given in Fig. S1.

*
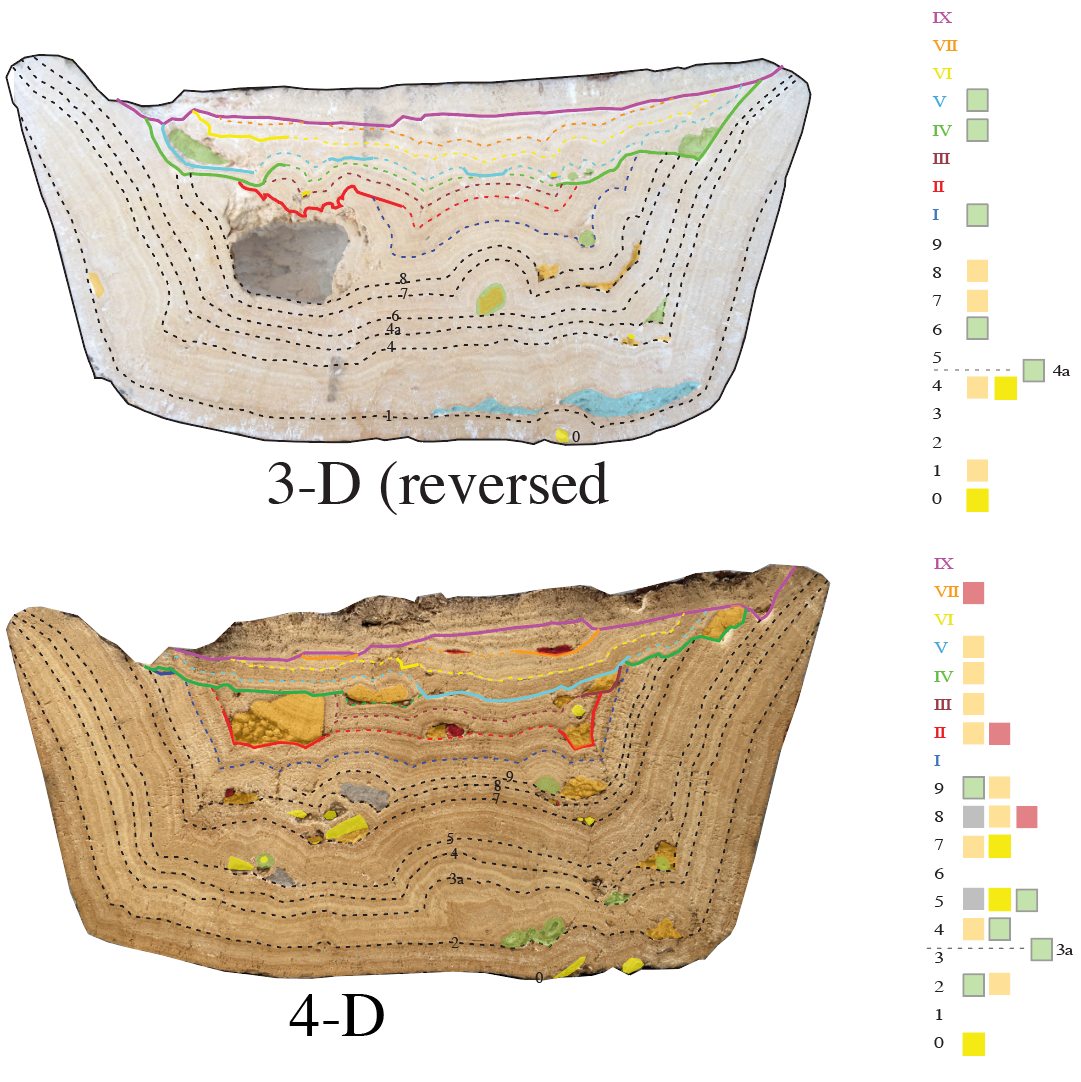
*

**Fig. S3, continued**

*
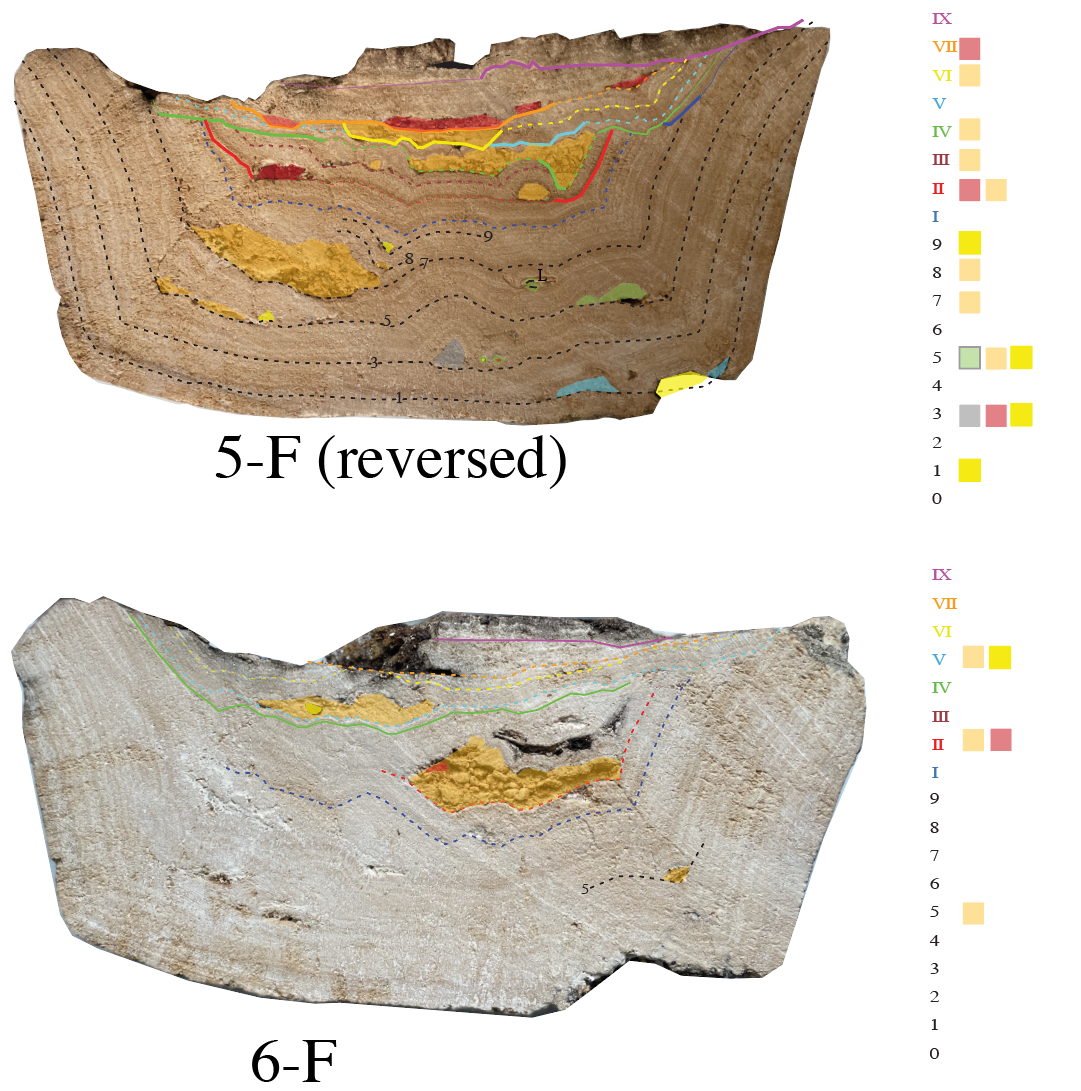
*

**Fig. S3, continued**

*
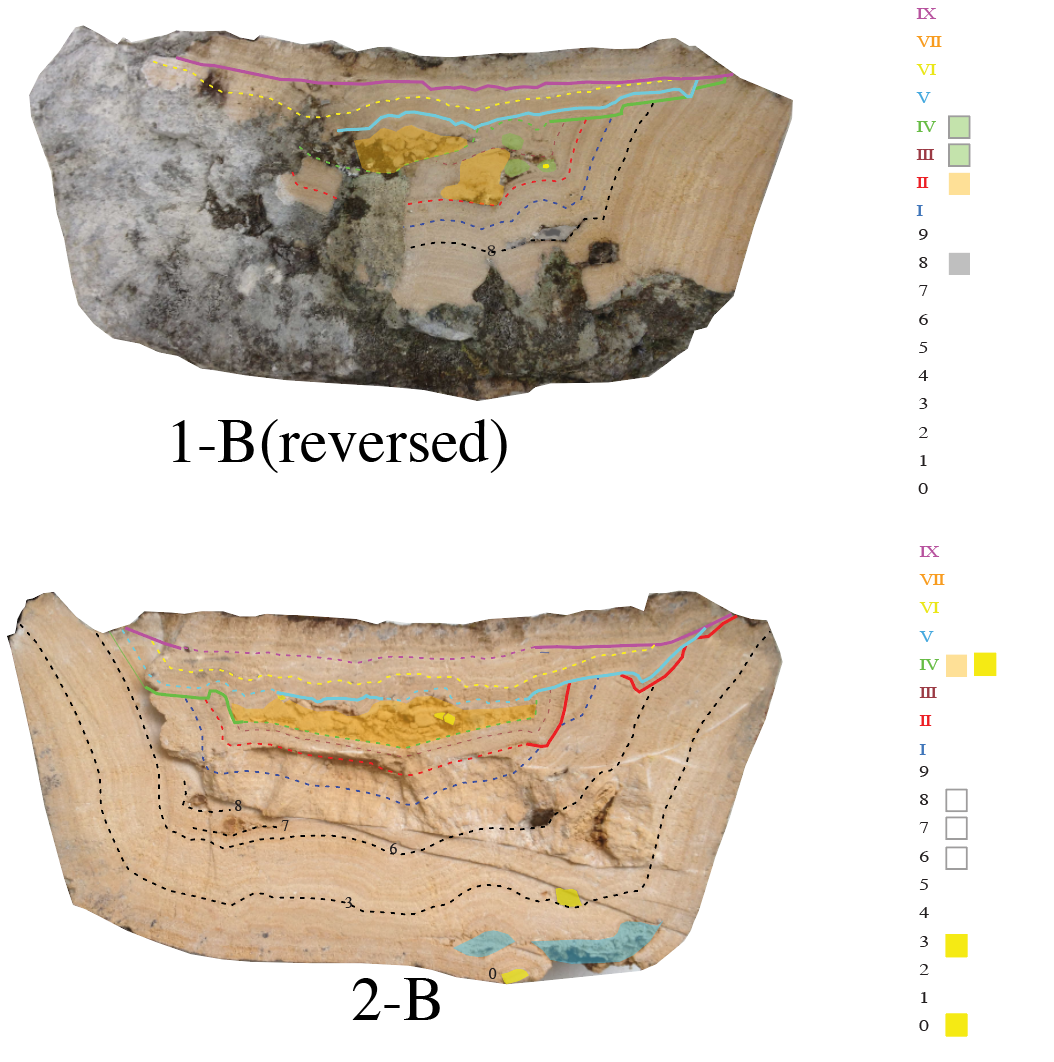
*

**Fig. S3, continued**

*
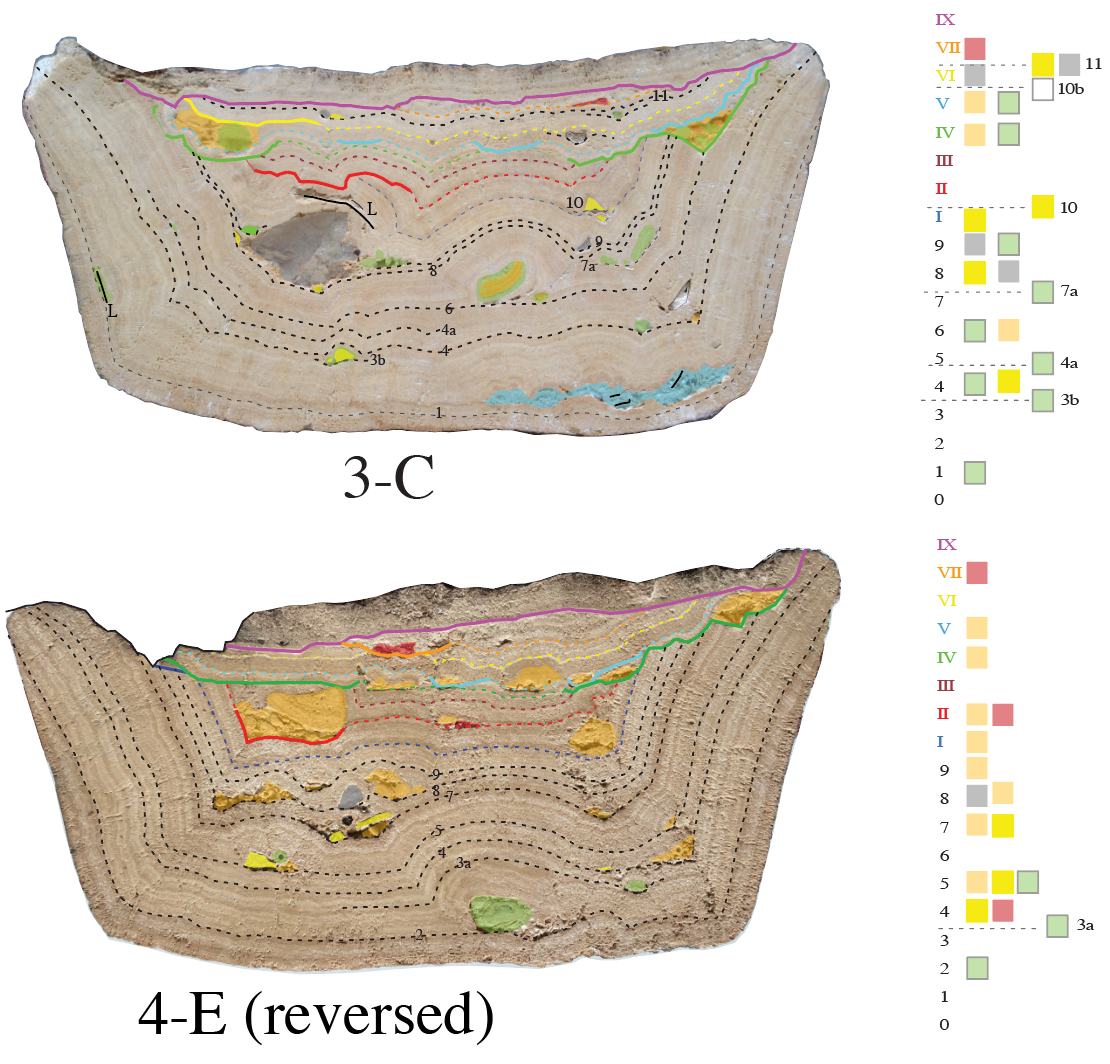
*

**Fig. S3, continued**

*
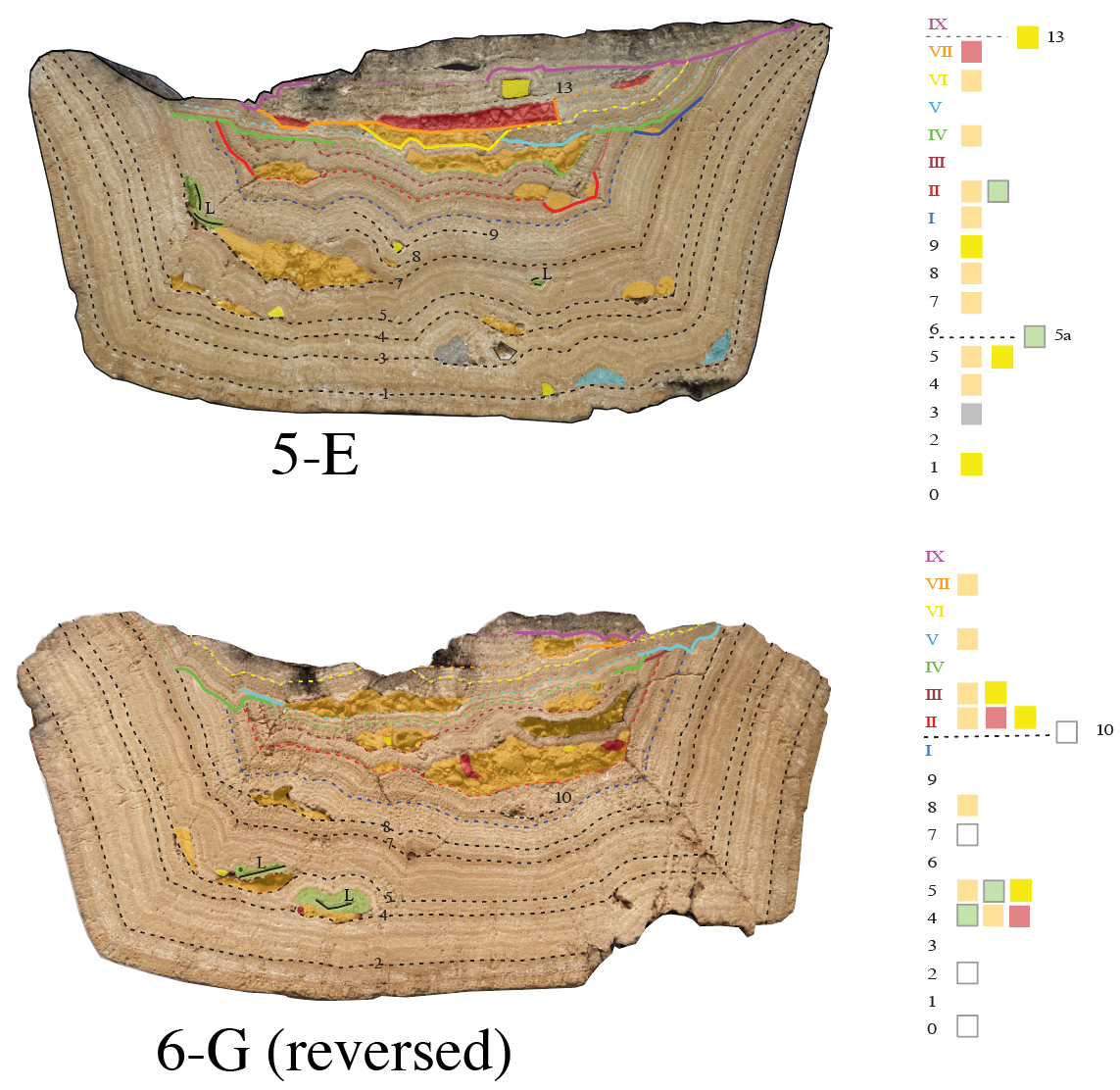
*

**Fig. S3, continued**

**Figure S4. Sample for analytical work**

*
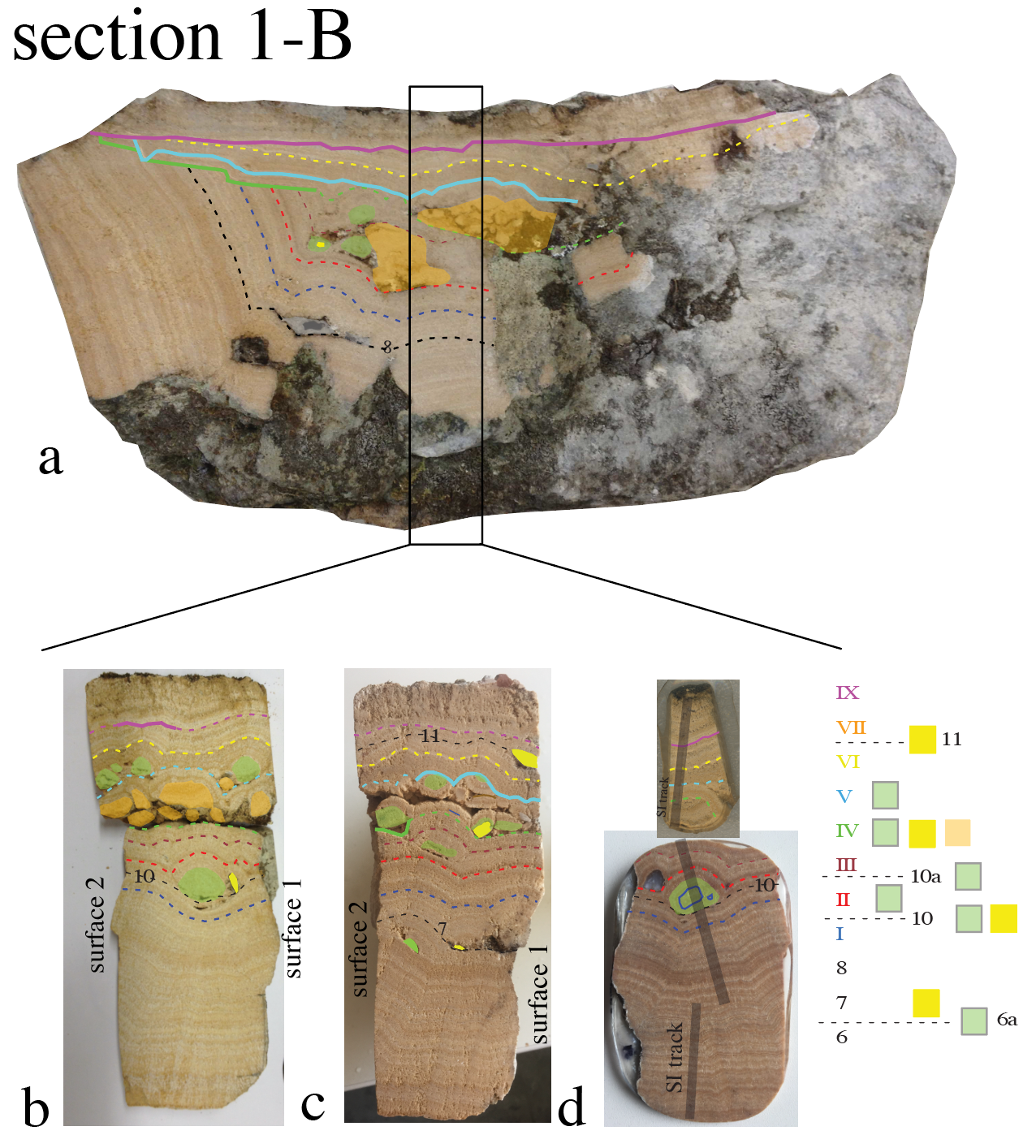
*

**Fig. S4**. (a) Slab B of the Nouailhac block with rectangle indicating position where sub-samples were taken for chemical and stable isotope analysis, and for preparation of thin sections. (b) and (c): slab faces on the left- and right-hand side of the cut in slab B. (c) was reversed to ease comparison. (d) fragments of slab B used for thin sections and for stable isotope analyses. An 8 mm diameter quartz pebble lies at top left on top of cleaning surface II. Such large pebbles are unlikely to be derived from the river source, since they would be accompanied by other gravel and sand and are unlikely to travel 17 km downstream without becoming covered by carbonate. Therefore, we suspect that this is material that was used for the preparation of mortar. Grey bars in (d) indicate micromilling tracks for stable isotope analyses presented in Fig. 4. Legend in Fig. S1

**Figure S5. Sidewall deposits**


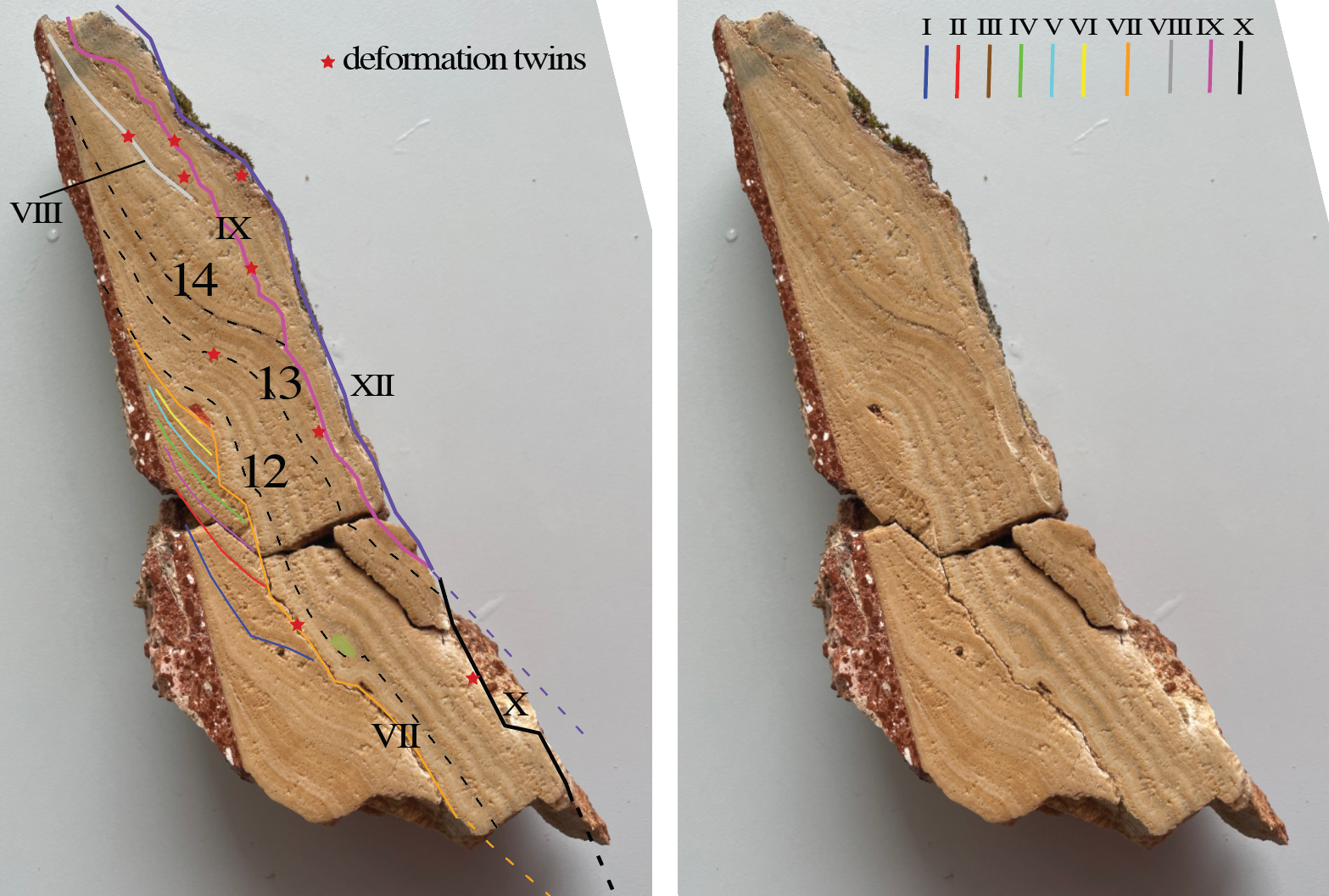


**Fig. S5**. Sidewall deposits from the NW side of the channel at Nouailhac in a marked and unmarked photograph. The position of the sample with respect to the main block is given in Fig. 2. Colored lines indicate cleaning surfaces. Sites where deformation twins were observed in corresponding thin sections are marked with red stars. Legend in Fig. S1

**Figure S6. Fragment types included on cleaning and fragment surfaces**


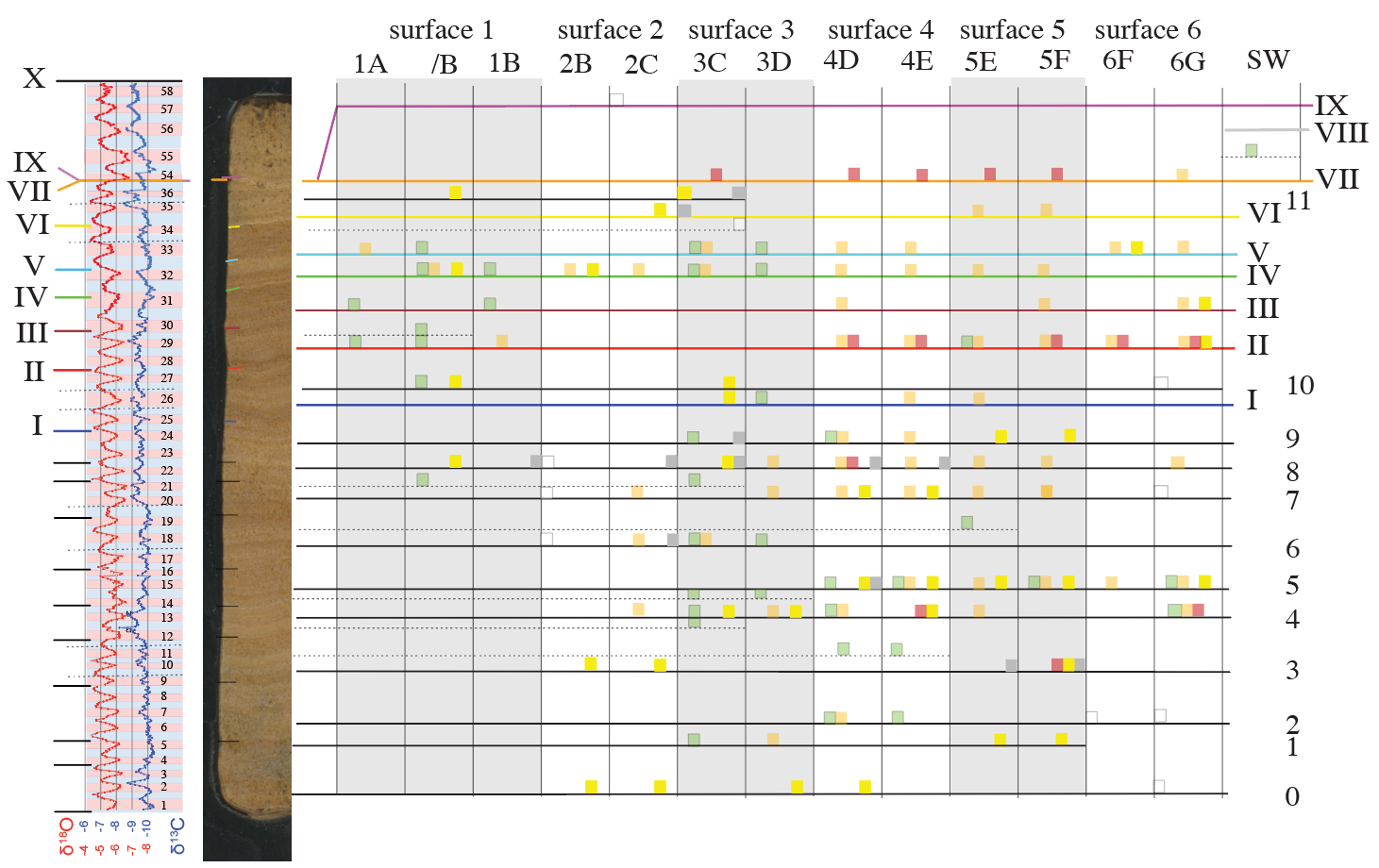


**Fig. S6.** Carbonate stratigraphy of the lower sequence as seen in the drill core, with stable isotope profile at left, and all observed fragment categories listed per slab face, given at right. The information on fragments is directly taken from the interpretation diagrams shown in Fig. S3. /B refers to oblique sections for analysis shown in Fig. S4. SW - sidewall deposits (Fig. S5). Color code legend given in Fig. S1.

**Figure S7. Microfabric observations**


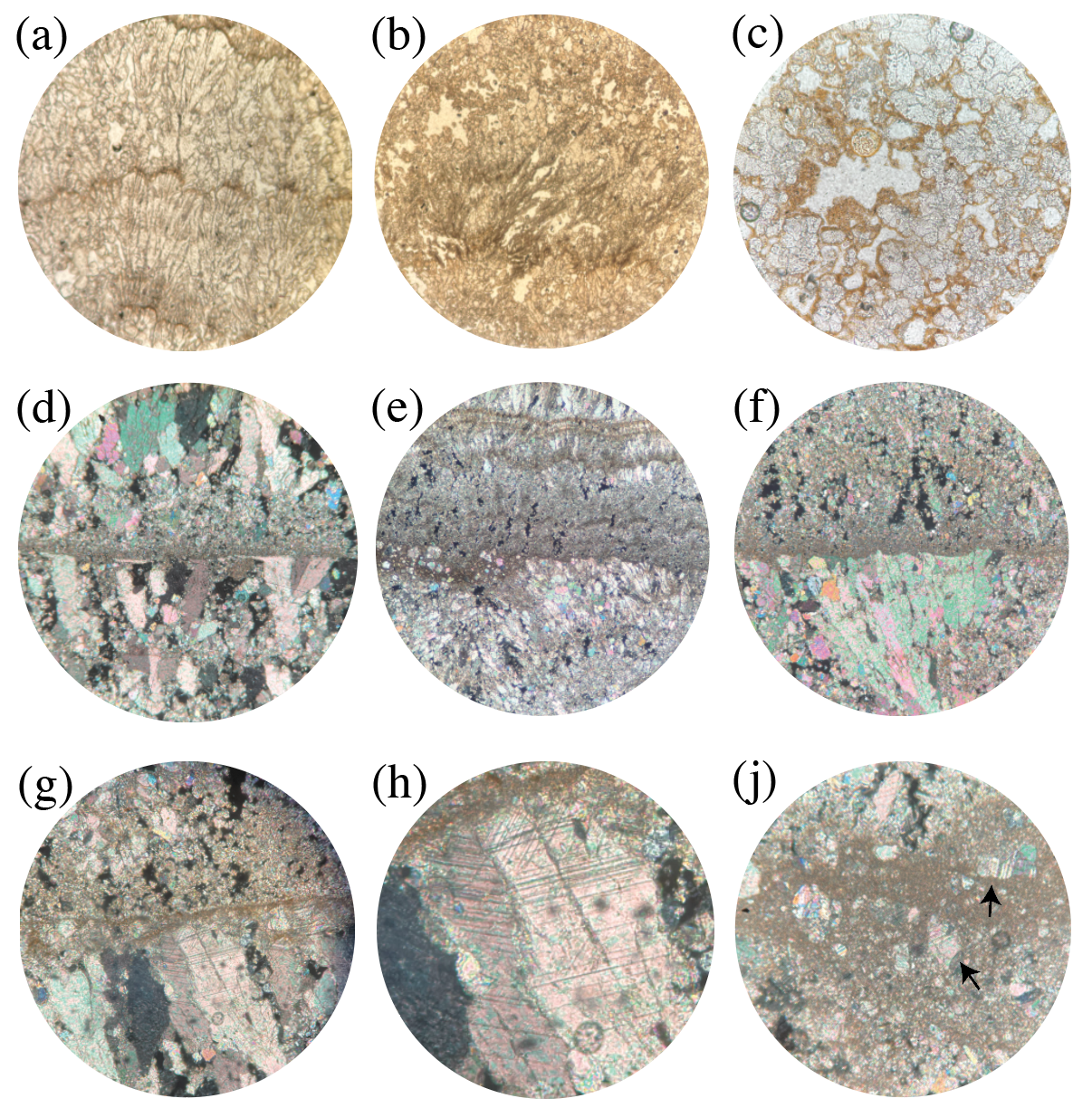


**Fig. S7**. Cahors carbonate microfabric. (a) typical aspect of the microsparitic fabric in most of the Cahors samples. Bundles of microsparitic calcite crystals growth are capped by a brown micrite band, followed by renewed microsparite growth; (b) typical porous micritic fabric of the top of the lower sequence (years 54-58 in Fig. 4), with dark strings of calcite crystals, nucleated on algal filaments; (c) top of the upper sequence (years 86-88 in Fig. 4) with truncated microsparite crystals capped by clay; (d-g) microfabric of unconformity surfaces in the lower sequence. These surfaces lie EW in the center of the image; (d) straight cleaning surface of transected microsparite crystals, covered by micrite; (e) truncated microsparite crystals (right) and brecciated calcite rubble (left): layering in the lower half is oblique to the unconformity surfaces; (f) micrite covering broken sparite crystals along an unconformity surface; (g) micrite covering damaged crystals with twins; (h) detail of (g) showing twins in the center; (j) breccia zone covering crushed crystals. Some fragments have deformation twins. Observations in (d)–(j) suggest that the unconformity surfaces are due to mechanical removal of carbonate. All images have youngest part at the top. (a)-(c) plain polarized light; (d)-(j) crossed polarized light. Width of view: a - 5mm; b-g 3mm; h,j 0.5mm.

**Figure S8. Flow chart of methods**

**
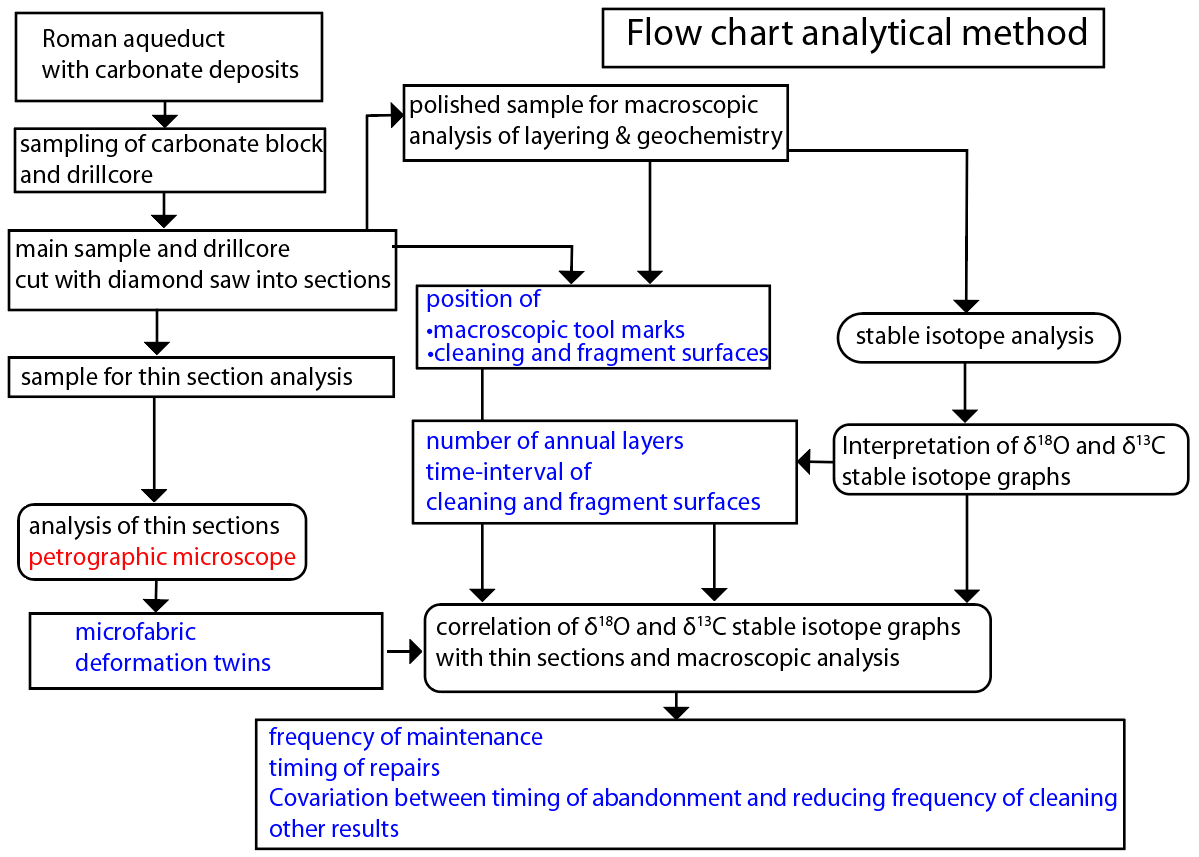
**

**Fig. S8**. Flow diagram for materials and methods in the text.

**Fig. S9. Diagram explaining carbonate deposition in aqueducts**


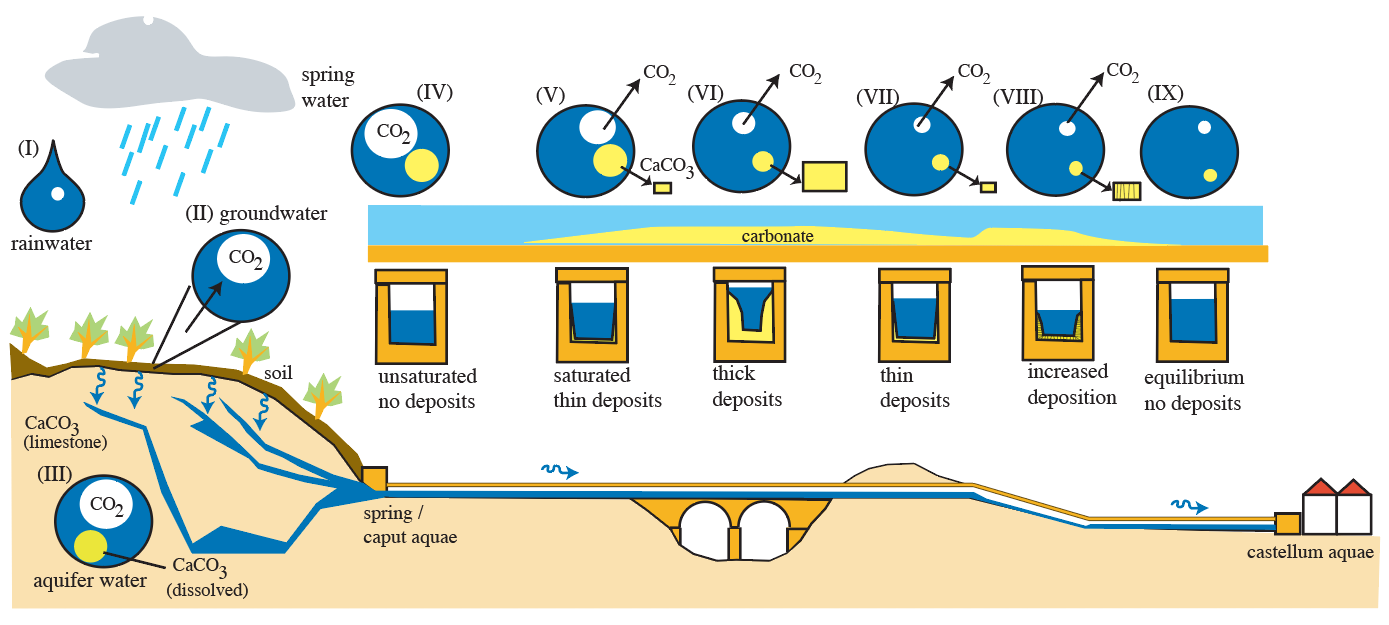


**Fig. S9**. Calcium carbonate deposition in water supply systems depends on the equilibrium of CO_2_ and Ca^2+^ dissolved in water. CO_2_ forms carbonic acid (H_2_CO_3_) in water, which dissociates into H^+^, HCO_3_^-^ and CO_3_^2-^ ions. CO_2_ concentration of rainwater is in equilibrium with CO_2_ in the atmospheric (I) but water is enriched in CO_2_ while travelling through the soil on its way to bedrock formations. The higher CO_2_ concentration in soil originates from the decay of organic matter and respiration of plant roots (II). Rainwater that penetrates the soil takes up this extra CO_2_ to form additional carbonic acid, which in an aquifer can dissolve CaCO_3_ if the bedrock is limestone or marble and can take up calcium ions from other rocks (III). When water thus enriched in Ca- ions and carbonic acid exits at a spring (IV), it will start to degas the excess CO_2_ to return to original atmospheric levels (V). Dissolved calcium carbonate will then precipitate on any surface in contact with water as CaCO_3_, in the form of the minerals calcite or aragonite. In most cases, some CO_2_ should degas for water to become saturated in CaCO_3_ before its deposition can start (IV). In practice, this means that deposition usually starts at some distance downstream, and hence almost no carbonate deposition can be seen in the first parts of many aqueducts (V). Carbonate deposition rate may then increase downstream to some maximum (VI), after which it decreases when water approaches an equilibrium concentration of CO_2_ with the atmosphere (VII). However, deposition rate may be enhanced at or shortly after parts of the system where water turbulence increases, such as in steep sections or drop shafts, or curves in the water line (VII). Once CO_2_ concentration in aqueduct water reflects that of the atmosphere again, e.g. in the final sections of long aqueducts, no CaCO_3_ deposition takes place (IX).
